# Probiotics, prebiotics, and synbiotics for patients with autism spectrum disorder: a meta-analysis and umbrella review

**DOI:** 10.3389/fnut.2023.1294089

**Published:** 2023-12-11

**Authors:** Fakher Rahim, Karlygash Toguzbaeva, Nameer Hashim Qasim, Kenesh O. Dzhusupov, Abzal Zhumagaliuly, Rabiga Khozhamkul

**Affiliations:** ^1^College of Health Sciences, Cihan University Sulaimaniya, Sulaymaniyah, Iraq; ^2^School of Public Health, Asfendiyarov Kazakh National Medical University, Almaty, Kazakhstan; ^3^Cihan University Sulaimaniya Research Center (CUSRC), Cihan University – Sulaimaniya, Kurdistan Region, Suleymania, Iraq; ^4^Head of Public Health Department, International Higher School of Medicine, Bishkek, Kyrgyzstan; ^5^Department of Biostatistics and Basics of Research, Asfendiyarov Kazakh National Medical University, Almaty, Kazakhstan

**Keywords:** autism spectrum disorder, probiotics, prebiotics, synbiotics, randomized controlled trials

## Abstract

**Background and objective:**

The potential impact of gut health on general physical and mental well-being, particularly in relation to brain function, has led to a growing interest in the potential health advantages of prebiotics, probiotics, and synbiotics for the management of ASD. A comprehensive meta-analysis and systematic review was conducted in order to evaluate the effectiveness and protection of many drugs targeted at manipulating the microbiota in the treatment of ASD.

**Methods:**

The present study employed a comprehensive examination of various electronic databases yielded a total of 3,393 records that were deemed possibly pertinent to the study. RCTs encompassed a total of 720 individuals between the ages of 2 and 17, as well as 112 adults and participants ranging from 5 to 55 years old, all of whom had received a diagnosis of ASD.

**Results:**

Overall, 10 studies reported Autism-Related Behavioral Symptoms (ARBS). Regarding the enhancement of autism-related behavioral symptoms, there wasn’t a statistically significant difference between the intervention groups (combined standardized mean difference = −0.07, 95% confidence interval: −0.39 to 0.24, *Z* = 0.46, *p* = 0.65). We observed that in the patients with ASD treated with probiotic frontopolar’s power decreased significantly from baseline to endpoints in beta band (Baseline: 13.09 ± 3.46, vs. endpoint: 10.75 ± 2.42, *p* = 0.043, respectively) and gamma band (Baseline: 5.80 ± 2.42, vs. endpoint: 4.63 ± 1.39, *p* = 0.033, respectively). Among all tested biochemical measures, a significant negative correlation was found between frontopolar coherence in the gamma band and *TNF*-α (*r* = −0.30, *p* = 0.04).

**Conclusion:**

The existing body of research provides a comprehensive analysis of the developing evidence that indicates the potential of probiotics, prebiotics, and synbiotics as therapeutic therapies for ASD. Our findings revealed that those there was no significant effect of such therapy on autism-related behavioral symptoms, it has significant effect on the brain connectivity through frontopolar power in beta and gamma bands mediated by chemicals and cytokines, such as *TNF*-α. The psychobiotics showed no serious side-effects.

## Introduction

Autism Spectrum Condition (ASD), a developmental condition, significantly influences people’s social interactions, behavior, and learning (1). While the diagnosis of this condition is possible at any age, its symptoms often become apparent within the first 2 years of life due to its inherent developmental characteristics. ASD has been seen to impact individuals from diverse ethnic, racial, and socioeconomic backgrounds. It is still unclear what causes autism spectrum disorder, most likely arising from a complex interplay of genetic and environmental influences (2–5). Parents and families have significant challenges when dealing with a kid who has been diagnosed with ASD since the disorder’s profound and wide-ranging deficits give rise to many complexities in providing care (6). In the last three decades, there has been a notable increase in the condition’s occurrence, leading to substantial research efforts to comprehend its biochemical and genetic markers (7). Nevertheless, there is a scarcity of research examining the intricate relationship between the symptoms of the condition and the dynamics within the family unit. While a considerable body of research has been dedicated to examining the difficulties encountered by these children, there has been minimal exploration of the particularities surrounding their caregiving contexts.

Unfortunately, there is no known remedy for ASD; however, various therapies have been devised and examined, primarily focusing on young children. The primary objective of these therapies is to mitigate symptoms, improve cognitive capabilities, strengthen daily life skills, and maximize social functioning among persons (8). The current body of knowledge about effective treatment approaches for individuals with ASD who are older children and adults is constrained. Although some study has been conducted on social skills groups for older children, the available data supporting their effectiveness still needs to be improved (9).

Treatment techniques with the potential to enhance outcomes throughout adulthood need to be evaluated, and this can only be done with further research. It is essential to provide services that support persons with ASD in their pursuit of education, vocational training, employment, housing, transportation, healthcare, daily functioning, and active participation in the community (10). The prompt identification and timely intervention of ASD in youngsters might provide substantial advantages, facilitating their ability to surmount several obstacles. Hence, it is essential for parents to proactively seek assistance from rehabilitation facilities upon detecting any signs of developmental delays or to meet with professionals in pediatric neurology and child and adolescent psychiatry. According to reference (11), the timely implementation of interventions may effectively minimize a significant proportion (ranging from 90 to 95%) of these concerns.

A range of therapeutic alternatives is accessible, including applied behavior analysis, social skills training, occupational therapy, physical therapy, sensory integration therapy, and the employment of assistive technologies (12). The treatments discussed in this context may be broadly classified into behavioral and communication techniques, dietary measures, medicine, and complementary and alternative therapies (13). Probiotics have garnered considerable interest within the field of nutrition. Live microorganisms provide several health advantages, a few of which will be further examined in subsequent sections of this article. In contrast, prebiotics, produced from indigestible carbohydrates, particularly fiber, function as a source of sustenance for the advantageous gut bacteria, specifically probiotics (14).

Moreover, a complete evaluation of the existing literature via an umbrella review reveals a scarcity of comprehensive meta-analyses investigating the simultaneous efficacy of probiotics, prebiotics, and synbiotics for patients diagnosed with ASD. Despite a few meta-analyses, the scope of these analyses is restricted due to the inclusion of only a small number of papers for pooled analyses (15–22). For this reason, it’s crucial to expand the scope of the literature review to incorporate additional studies on the advantages of combining probiotics, prebiotics, and synbiotics for children with ASD. This study aims to collecting evidence on the efficacy of probiotic, prebiotic, and synbiotic therapy plans. It will aid in formulating well-informed guidelines and procedures for implementing these therapies within the framework of ASD care. The task at hand also necessitates investigating essential implementation details.

## Methods

The standards for the Preferred Reporting Items for Systematic Reviews and Meta-Analyses (PRISMA) statement were used to make this systematic review and meta-analysis (23).

### Search strategy

We conducted a comprehensive search across widely recognized indexing databases, which included CNKI, PubMed/MEDLINE, Embase, Web of Sciences, Scopus, and the Cochran library. Our search strategy employed broad search terms encompassing various expressions including Search: ((((((autistic traits[Title/Abstract]) OR (Asperger disorder[Title/Abstract])) OR (Asperger syndrome[Title/Abstract])) OR (autistic disorder[Title/Abstract])) OR (autism[Title/Abstract])) OR (autism spectrum disorder[Title/Abstract])) AND ((((probiotics[Title/Abstract]) OR (prebiotics[Title/Abstract])) OR (synbiotics[Title/Abstract])) OR (psychobiotics[Title/Abstract])). This search covered the period from January 1, 1980, to August 15, 2023, with no language restrictions applied. Furthermore, we extended our search by screening the references of selected studies and pertinent review articles. This extra check was done to find relevant studies that did not come up in the primary database searches. To facilitate efficient organization and management of the retrieved references, we established a bibliographical database using EndNote X7. To ensure accuracy and consistency, two authors (FR and KD) independently assessed each paper for eligibility. Any discrepancies were resolved through consultation with third author (KT).

### Study selection

Our study encompassed trials characterized by the following attributes:

*Study Type*: disciplinary trials involving the diagnosis of autism spectrum disorder were scrutinized exclusively, Asperger disorder, Asperger syndrome, or autistic disorder utilizing the widely accepted Randomized Controlled Trial (RCT) design.

*Participants*: our research was limited to individuals between the ages of 1–60 who were diagnosed with autism spectrum disorder (ASD), Asperger disorder, autistic disorder, or autism spectrum condition.

*Intervention*: we scrutinized interventions involving probiotics, prebiotics, and symbiotics alone or in conjunction with other nutritional supplements, contrasting against a placebo.

*Outcomes*: the outcome measures include primary outcome as Effects of Probiotics, prebiotics, and synbiotics on Autism-Related Behavioral Symptoms of Children with ASD. To assess Autism-Related Behavioral Symptoms, included studies mostly used the Aberrant Behavior Checklist. The Aberrant Behavior Checklist (ABC) (24, 25) consists of 58 questions asked of parents on a 0–3 scale, broken down as follows: (1) irritability (15 questions covering agitation, aggression, and self-injury); (2) social withdrawal; (3) stereotypes; (4) hyperactivity; and (5) improper speech (4 items) (26). The ABC is commonly used in ASD RCTs (27). The included studies’ mean and standard deviation (SD) for the transformation in outcome measures from pre- to post-intervention for ASD-related conduct disorder (henceforth referred to as “change in score”).

Secondary outcomes were biochemical and clinical parameters, as well as change in electroencephalogram (EEG). Neurological and psychiatric examinations were included in the clinical evaluation, in addition to a standardized assessment of gastrointestinal symptoms using the GSI (28); autism severity through ADOS-2 (29), Childhood Autism Rating Scale (CARS) (30), and Social Communication Questionnaire (SCQ) (31); limited and repetitive actions utilizing the Revised Repetitive Behavior Scale (RBSR) (32); screening for emotional, behavioral, and social issues with the Child Behavior Checklist (CBCL) (33); improvements in one’s mental faculties as measured by means of the Griffiths Mental Development Scales-Extended Revised (GMDS-R) (34); improvement in adaptive skills as measured by the Vineland Adaptive Behavior Scales-II (35); language abilities can be assessed using the McArthur-Bates Communicative Development Inventories (CDI) (36).

Excluded from our analysis were trials meeting the following criteria:

- Studies lacking precise and distinct inclusion and exclusion criteria.
- Outcomes that needed to be explicitly defined or elucidated.
- Trials lacking a controlled study design.
- Pregnant or breastfeeding women participants.
- Preclinical investigations using experimental animals.

In instances where several papers presented identical or overlapping data, preference was given to articles with lengthier intervention durations or larger sample sizes, incorporating them into our study.

### Gastrointestinal and autism-related symptoms

We used a 7-point Likert scale to collect information about GI symptoms by administering a customized form of the Gastrointestinal Symptom Rating Scale (GSRS) (37) in the five areas of tummy trouble (ache, reflux, indigestion, loose stools, and bowel obstruction). Using the Bristol Stool Form scale, we also collected Daily Stool Records (DSR) for a total of 14 days (1 = very hard, 7 = liquid). Parent Global Impressions-III (PGI-III), Childhood Autism Rating Scale (CARS), Aberrant Behavior Checklist (ABC), Social Responsiveness Scale (SRS), and Vineland Adaptive Behavior Scale II were all used to evaluate symptoms associated with autism, as they had been in the previous study (VABS-II). About 2 years after treatment ended, parents evaluated their child using the GSRS, DSR, PGI-III, ABC, SRS, and VABS-II, and the evaluation was conducted by the same professional evaluator who had previously conducted the CARS evaluation.

### Data extraction

At the outset, a pair of researchers (referred to as FR and KD in this study) conducted an initial screening of the gathered literature. This sifting involved evaluating the abstracts and titles to determine which works met our predetermined criteria. Subsequently, these selected works underwent a thorough assessment by the same researchers. They individually reviewed the full-text articles and extracted a range of data points, encompassing fundamental participant characteristics, sample sizes, particulars of interventions, comparative measures, intervention durations, evaluations of behavioral symptoms associated with autism, scores of GI symptoms, and other relevant details.

Any disparities between the assessments conducted by these two researchers were resolved either by double check or discussion. Alternatively, a third reviewer (referred to as KT) was consulted.

### Study quality assessment

Following the guidelines specified in the PRISMA statement, the evaluation of discrimination hazards in randomized controlled trials, also known as RCTs, and crossover trials involved a thorough assessment of seven crucial factors: (1) the generation of random sequences; (2) the concealment of allocation; (3) the blinding of participants and personnel; (4) the blinding of outcome assessment; (5) the handling of incomplete outcome data; (6) the elimination of chosen reporting; and (7) the identification of any additional potential sources of bias. Each of the bias-related characteristics was classified into one of three categories: low risk, uncertain risk, or high risk.

### Umbrella review

We conducted an umbrella analysis by conducting systematic searches in databases such as MEDLINE, Embase, the Cochrane Central Register of Controlled Trials, and more than 30 other sources. This review followed the JBI systematic review methodology. The Grading of Recommendations, Assessment, Development, and Evaluation approach was used to assess the certainty of evidence. Covidence was used to carry out the selection process (Melbourne, Australia).

For eligibility determination, two independent reviewers evaluated titles and abstracts. The inclusion of studies was confirmed through a full-text review to ensure alignment with the selection criteria. All screening decisions were meticulously documented and are outlined in this report, accompanied by a comprehensive list of studies that were excluded. Eligible studies underwent a thorough appraisal by one reviewer and were cross-verified by a second reviewer. The AMSTAR-2 was used to evaluate systematic reviews and meta-analyses (38).

### Data analysis

The assessment of potential bias in randomized controlled trials (RCTs) and crossover studies was conducted using Review Manager 5.3. Review Manager 5.3 was used for conducting all meta-analyses and generating visual representations. Furthermore, the study used STATA/SE software (version 15.1) and the “Meta-Analysis” package. To assess the changes in scores based on behavioral associated symptoms with autism spectrum disorder (ASD) between the first assessment and the final evaluation (referred to as “change in score” afterward), the average values and standard deviations (SDs) were obtained from both the intervention and control groups in the studies included in the analysis.

When the original sources or the writers failed to include direct standard deviations (SDs) for score changes, SDs were approximated by using the baseline and endpoint score SDs, in conjunction with the correlation value of 0.5, as recommended in the Cochran handbook’s recommended formula. The researchers then used Hedges’ technique to compute the standardized mean difference (SMD) and 95% confidence intervals (CIs) to assess the magnitude of the impact.

To assess the heterogeneity across studies, we used the *I*^2^ statistical and the value of *p* derived from Cochran’s *Q* test. In this study, *I*^2^ values less than 25% were indicative of low heterogeneity, while values ranging from 25 to 50% were considered as moderate heterogeneity. On the other hand, values beyond 50% were classified as high heterogeneity. Utilizing a significance threshold of *p* < 0.05, found evidence of statistically significant heterogeneity. A fixed-effects model was selected if the *I*^2^ value was below 50%, whereas the random-effects model was utilized if the *I*^2^ value had been equal to or higher than 50%.

The Egger and Begg tests were performed in order to assess publication bias. The investigation of causes of heterogeneity included the examination of subgroups, taking into account several characteristics such as the country in which the research was conducted, the scales that were employed, the methods of intervention, the length of the intervention, and the kinds of studies, all of which were considered as possible criteria for subgroup classification.

To ensure the robustness of the results, sensitivity analyses were conducted by excluding one research and then redoing the meta-analysis. For all analyses conducted, a significance threshold of *p* < 0.05 was used for two-sided testing.

## Results

### Features shared by included studies

**Figure 1:**
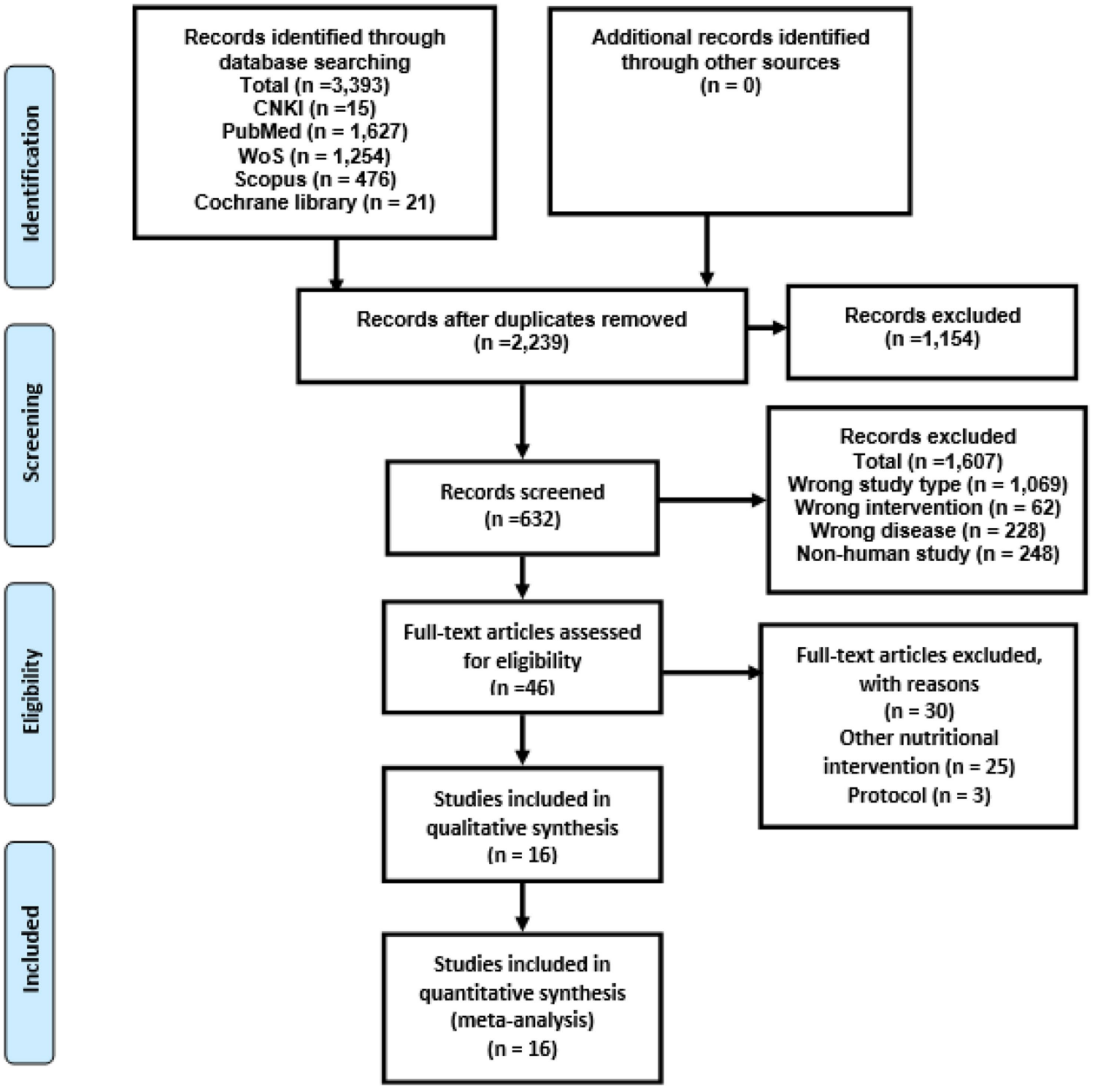
PRISMA flowchart for included studies.

The PRISMA flowchart guided the study selection procedure, which included several stages (Figure 1). At first, 3,393 results were found after searching multiple databases online. Among these, 1,154 records were identified as duplicates and subsequently removed. Following this, a thorough evaluation of the titles and abstracts of the remaining 2,239 unduplicated articles led to the exclusion of 1,607 articles that did not align with the criteria. Consequently, 46 reports remained for a comprehensive full manuscript review. Upon conducting a detailed review of the full articles, 16 trials met our predefined inclusion criteria. These 16 trials were consequently selected for incorporation into the present systematic review and subsequent meta-analysis. For a comprehensive overview of the characteristics of these 16 randomized controlled trials (RCTs), please refer to Table 1 (7, 39–52, 54–56). Overall, 720 children with mean ages 2 to 17 years (7, 39, 41–52, 54, 56), 112 adults and participants aged 5 to 55 years with ASD. Of 16 included studies, 15 used probiotics and one used prebiotics. Out of 16, seven were from the USA (7, 40–42, 47, 50, 52), four from China (43–45, 49), two from the UK (51, 54), one from each Italy, Taiwan, and Egypt (39, 46, 48).

**Table 1:** Qualities of included controlled experiments.

Study, year (ref.)	Country	Total sample	Intervention of experimental group (dose)	Target population	Male/Female	Duration in weeks	Mean age (Rang)	Outcomes
Billeci et al. (39)	Italy	63	De Simone’s probiotics included in the mix are: *Lactobacillus casei*, *Bifidobacterium breve*, *Bifidobacterium longum*, *Bifidobacterium infantis*, and *Streptococcus thermophilus*.	ASD	35/11	6	46.56 months ± 13.92 (18–72 months)	ADOS CSS, ADI-R, SCQ, RBS-R, General Quotient, Developmental ret., VABS-II, Linguistic Level, CBCL 1, PSI, GSI Severity Index, CARS, TNF-α, CCL2, Leptin, Resistin, PAI-1
Schmitt et al. (40)	USA	15	Probiotics: SB-121, a combination of *L. reuteri*, Sephadex^®^ (dextran microparticles), and maltose	ASD	15/0	4	20.0 ± 3.05 (15–45 years)	Vineland factors, Oxytocin, TNF-α, and HS-CRP
Simmons et al. (41)	USA	69	Probiotics: Vivomixx	ASD	57/12	12	7.8 ± 2.6 years	ATEC GHI ABC
Kong et al. (42)	USA	35	Probiotics: *Lactobacillus plantarum*	ASD	26/9	6	10.3 (3–20 years)	serum OXT, MBP, GFAP, S100B, IL-1β, GSI, CGI
Li et al. (43)	China	41	Probiotics: Lactobacillus and Enterococcus Powder	ASD	–	3	3–6 years	Applied behavior analysis (ABA)
Santocchi et al. (44)	China	85	Probiotic supplement, DSF	ASD	71/14	24	4.13 ± 1.0 years	ADOS-CSS, VABS-II, GMDS-ER, 6-GSI, ATEC
Wang et al. (45)	China	50	Prebiotics: *Lactobacillus plantarum* + FOS	ASD	–	3	3–9 years	Dopamine metabolism disorder, hyperserotonergic state (increased serotonin), and the presence of acetic acid, propionic acid, and butyric acid
El-Alfy et al. (46)	Egypt	100	Probiotics: Lacteol Fort	ASD	–	12	2–10 years	ATEC, 6-GSI
Arnold et al. (47)	USA	13	The eight probiotic species found in VISBIOME are primarily Lactobacillus and Bifidobacterium	ASD	9/4	19	3–12 years	ADOS2, PedsQL GI, PRAS-ASD, ABC, SRS, CSHQ, PSI
Kang et al. (7)	USA	18	Probiotics: *Lactobacillus plantarum*	ASD	–	18	7–17 years	Vineland factors, ADOS2, PedsQL GI, PRAS-ASD, ABC, SRS, CSHQ, PSI
Liu et al. (48)	Taiwan	80	Probiotics: *Lactobacillus plantarum* PS128	ASD	–	4	10.01 ± 2.34 years	CGI-I, SRS, CBCL, SNAP-IV
Niu et al. (49)	China	37	Applied behavior analysis (ABA) training in combination with probiotics	ASD	25/12	4	4 (3–8 years)	ATEC, GI score
Sanctuary et al. (50)	USA	16	Probiotics: *Bifidobacterium infantis* + BCP	ASD	11/5	20	6.8 ± 2.4 (2–11 years)	ABC, GIH
Grimaldi et al. (51)	UK	41	Prebiotic: Bimuno^®^ galactooligosaccharide (B-GOS^®^) prebiotic intervention	ASD	31/10	6	7 (4–11 years)	ATEC, EQ-SQ, SCAS-P
Kang et al. (52)	USA	18	Probiotics: *Lactobacillus plantarum*	ASD	–	8	7–16 years	Vineland factors, ADOS2, PedsQL GI, PRAS-ASD, ABC, SRS, CSHQ, PSI
Kałużna-Czaplińska and Błaszczyk (53)	Poland	22	Probiotics: *Lactobacillus acidophilus*	ASD	20/2	8	5.6 ± 1.6	Changes in DA/LA
Parracho et al. (54)	UK	17	Probiotics: *Lactobacillus plantarum* WCFS1	ASD	–	12	3–16 years	DBC

### Evaluation of bias and quality in individual study assessments

**Figure 2:**
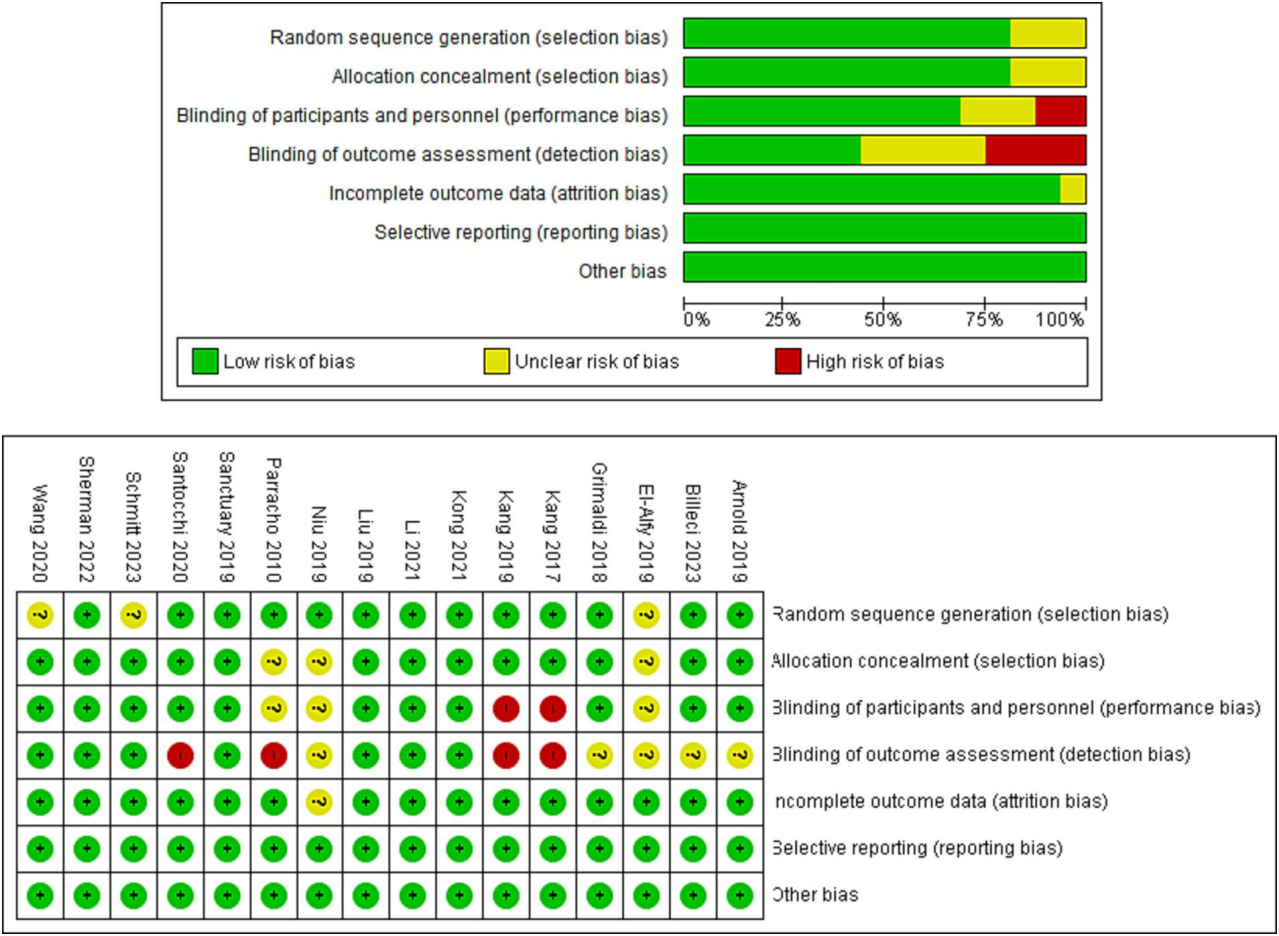
Risk of bias assessment in included randomized controlled trials (RCTs).

The analysis of 16 cases revealed that 93.75% (15/16) showing the investigations provided sufficient documentation of randomized sequence creation. However, the other two studies exhibited ambiguity in this particular area. All of the studies yielded data about the concealment of allocation. Out of the total number of trials examined, 68.75% (11 out of 16) were found to have successfully adopted double-blinding for outcome assessors. However, it is worth noting that blinding procedures were not conducted in four particular studies, namely trials (7, 44, 52, 54). The findings from most studies indicated a little risk of bias when it came to the blinding of participants and key research employees. However, it should be noted that two experiments demonstrated a significant potential for bias about this matter. Moreover, it was observed that all studies had a minimal likelihood of bias about inadequate outcomes knowledge and selective result reporting. The data is graphically presented in Figure 2, which includes a graph (A) illustrating the risk of bias and a summary (B) outlining the risk of bias for the RCTs (randomized controlled trials) that were included in the study.

### Primary outcome evaluation of probiotic, prebiotic, and synbiotic effects on autism spectrum disorder-related behavioral symptoms

**Figure 3:**
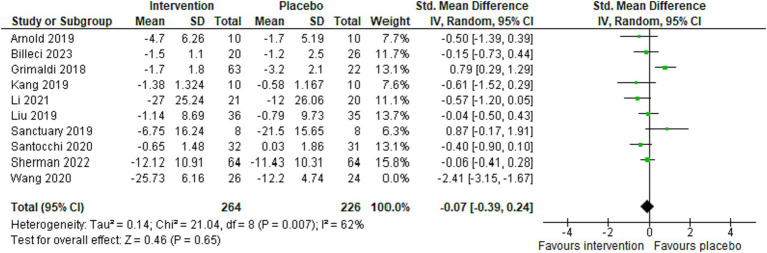
Forest plot illustrating the impact of psychobiotics on enhancing autism-related behavioral symptoms in the intervention vs. placebo groups.

Overall, 10 studies reported Autism-Related Behavioral Symptoms (ARBS) (7, 39, 41, 43–45, 47, 48, 50, 51). We used the random-effects model due to high heterogeneity between studies (*p* = 0.007, *I*^2^ = 62%). Regarding the enhancement of autism-related behavioral symptoms, the results of the intervention group were not significantly different from the control group (combined standardized mean difference = −0.07, 95% confidence interval: −0.39 to 0.24, *Z* = 0.46, *p* = 0.65) (Figure 3).

### Assessing secondary outcomes: effects of probiotics, prebiotics, and synbiotics on EEG, and biochemical and clinical parameters

Patients with ASD who took probiotics had a statistically significant reduction in beta band semantic similarity power between baseline and follow-up (Baseline: 13.09 ± 3.46, vs. endpoint: 10.75 ± 2.42, *p* = 0.043, respectively) also gamma spectrum (Baseline: 5.80 ± 2.42, vs. endpoint: 4.63 ± 1.39, *p* = 0.033, respectively) compared with no significant change in placebo group (39). Frontal asymmetry in individuals with ASD who were given probiotics showed a significant decrease between baseline and endpoints in delta band (Baseline: 0.029 ± 0.053, vs. endpoint: −0.024 ± 0.047, *p* = 0.032); while those on the placebo group saw a significant increase from baseline to endpoints in frontopolar asymmetry in the alpha band (Baseline: 0.022 ± 0.043, vs. endpoint: 0.077 ± 0.043, *p* = 0.03). The gamma-band power of frontopolar regions was positively correlated with the total number of RBS-R endorsements (*r* = 0.28, *p* = 0.04), which means that after taking probiotics, young children who had a lower RBS-R overall number had a lower frontopolar power in the gamma group. The beta and gamma frontopolar coherence results from VABS-II were positively correlated with one another (*r* = 0.37, *p* = 0.012 and *r* = 0.40, *p* = 0.007, respectively), so, those with ASD who scored lower on the VABS-II after taking probiotics exhibit greater beta and gamma frontopolar coherence. Frontopolar gamma coherence was found to have the strongest inverse correlation with *TNF*-α of any biochemical indicator tested. (*r* = −0.30, *p* = 0.04), resulted in greater frontopolar coherence in the gamma band after probiotic administration in ASD subjects with lower *TNF*-α levels at post-test.

### Subgroup analyses

It was found through country-specific subgroup analyses that no region showed statistically significant differences in the improved performance of assessments of behavioral symptoms related to ASD between the therapy and placebo groups (Table 2). There was also no statistically significant difference between the groups who received intervention and the groups who received a placebo when it came to the improvements in behavioral symptom severity affiliated to autism spectrum disorder (ASD) (Table 2).

**Table 2:** Subgroup analysis of autism-related behavioral symptoms by geographic region, intervention type.

Sub-grouped by	No. of trials	No. of participants	SMD	95% CI	*p*	*I*^2^ (%)	*p* for heterogeneity
Geographic region							
America Europe Asia	4 2 4	184 131 225	−0.11 0.33 −0.82	−0.6, 0.39 −0.58, 1.25 −1.71, 0.06	0.67 0.47 0.07	45% 82% 90%	0.14 0.002 <0.00001
Intervention type							
Probiotics Prebiotics	8 2	405 135	−0.19 −0.80	−0.42, 0.33 −3.93, 2.34	0.09 0.62	17% 98%	0.30 <0.00001

### Analyzing the impact of publication bias and variables

**Figure 4:**
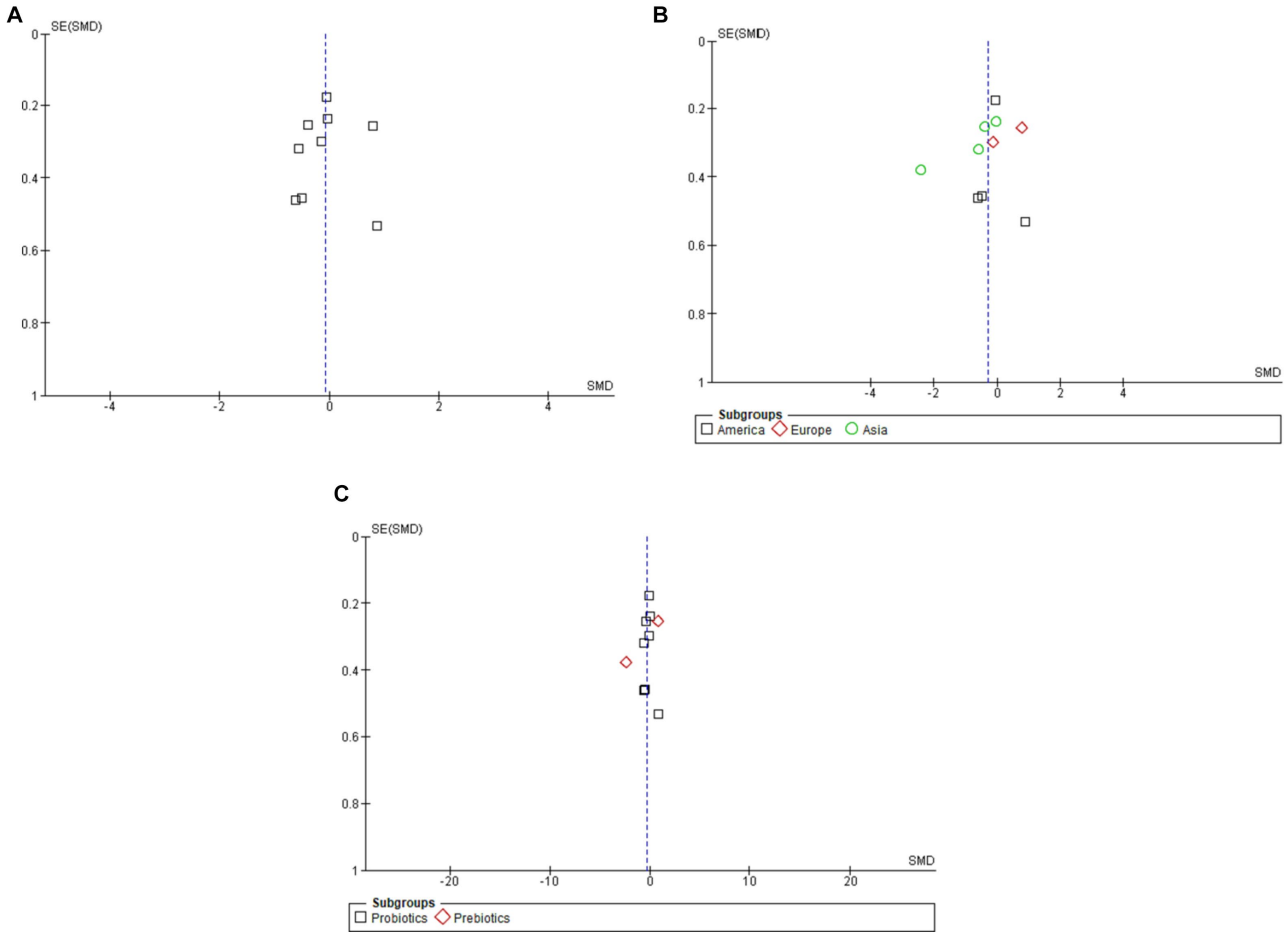
Funnel plots of overall **(A)**, subgroup analysis by geographic region **(B)**, and subgroup analysis by type of intervention **(C)**.

The total number of papers used in this meta-analysis was 10. Evidence of publication bias was sought using the methods established by Begg and Egger’s experimental studies and visual check of funnel plots for symmetry (Figure 4). These statistical tests indicated a little chance of editorial prejudice (*p* > 0.05). To test the robustness of the results, the seven publications include in the meta-analysis were subjected to a sensitivity analysis. Importantly, when individual research studies were removed, there was still little heterogeneity in the aggregate impact size. This further demonstrates the validity of the results of this meta-analysis.

### Harmlessness

There were no unexpectedly serious AEs, which was expected. Neither treatment-attributable nor gastrointestinal AEs were more common in the probiotic preparation than in the placebo group (47, 51). This further verifies the formulation’s proven safety profile.

### Umbrella review

Finally, we located eight small-sample systematic reviews and meta-analyses on the probiotics, prebiotics, and synbiotics for ASD. Results from 125 randomized controlled trials were analyzed for 41 pharmaceuticals and 17 dietary supplements. (*n* = 7,450 participants) teenagers and kids and 18 RCTs (*n* = 1,104) in adults that were conducted in several worldwide databases by Siafis et al. (20). He et al. (17) did a similar meta-analysis to examine if probiotics might ameliorate behavioral indicators in children with ASD. They found seven papers that supported this hypothesis. When investigating whether probiotics and prebiotics may reduce the intensity of symptoms of ASD in young ones, the complexity of gastrointestinal (GI) disorders, and the concomitant psychopathology in ASD, Song et al. (21) did a meta-analysis using just 3 clinical controlled trials. Only Ng et al. (19) analyzed eight clinical studies to determine the impact of prebiotics/probiotics on ASD. When it comes to treating core and co-occurring behavioral problems in people with ASD, 14 papers satisfied the inclusion criteria for a recent review by Tan et al. (22), in which they critically examine the available data on the effectiveness and efficacy of probiotics, prebiotic, synbiotic, and transplantation of feces microbiota treatments. Barbosa and Vieira-Coelho (16) tried to identify the functioning clinical proof that could possibly defend the use of probiotics or prebiotics in neurological patients and included 11 studies; Ligezka et al. (18) completed a literature review on the effects of the gut microbiota on the mental health of children and adolescents; 7 studies, along with RCTs and cohort studies, met eligibility requirements. Finally, Alanazi (15) conducted a meta-analysis of randomized, controlled studies to determine whether or not probiotics and vitamins are beneficial for people with ASD. Table 3 lists the specifics and features of these evaluations.

**Table 3:** Characteristics of included systematic review and meta-analyses.

Study, year (ref.)	Country	Total included	Intervention of experimental group (dose)	Study design	Duration in weeks	Target group	Outcomes
Siafis et al. (20)	Germany	18	Pharmacological and dietary-supplement	SR and MA	8–13 weeks	Children adolescents and adults	Medication for the primary symptoms should not be prescribed on a regular basis
He et al. (17)	China	10	Probiotics	SR and MA	4–12 weeks	Children	The influence of probiotics on children with ASD need to be studied in randomized controlled trials (RCTs) that adhere to rigorous trial guidelines
Song et al. (21)	China	3	Prebiotics and probiotics	SR and MA	4–24 weeks	Children	Future, more randomized controlled studies are needed
Ng et al. (19)	Singapore	8	Prebiotics and probiotics	SR	3–12 weeks	Children	Despite promising preclinical findings, prebiotics and probiotics have limited efficacy in ASD
Tan et al. (22)	Canada	14	Probiotics, prebiotics, synbiotics	SR	1–18 weeks	Children	Beneficial effects of probiotic, prebiotic in ASD
Ligezka et al. (18)	USA	7	Prebiotics	SR	3–12 weeks	Children adolescents	Research is needed to confirm whether or not gut dysbiosis
Barbosa and Vieira-Coelho (16)	Portugal	11	Prebiotics and probiotics	SR	4–18 weeks	Children	Findings in specific psychiatric disorders are encouraging
Alanazi (15)	Saudi Arabia	11	Prebiotics and supplements	SR	–	Children adolescents	Still lacks stronger evidence
Present study	Iraq	18	Probiotics, prebiotics, synbiotics	SR, MA, and UR	4–28 weeks	Children adolescents and adults	No beneficial effects of probiotic, prebiotic in ASD

### Risk of bias in included systematic reviews

We evaluated the potential bias in all the studies that were included in the analysis. The outcomes of this bias assessment are presented in Table 4. To ensure that all relevant studies were included, systematic reviews should ask specific questions, develop thorough search strategies, and employ a variety of resources. The methods used to standardize the extraction of data and pool findings from multiple studies were also solid.

**Table 4:** Methodological quality evaluation of the included systematic reviews.

Study ID	Q1	Q2	Q3	Q4	Q5	Q6	Q7	Q8	Q9	Q10	Q11
Siafis et al. (20)	Y	Y	Y	N	Y	Y	Y	Y	N	N/A	Y
He et al. (17)	Y	N	Y	N	Y	Y	Y	Y	N	Y	N
Song et al. (21)	Y	u	Y	N	Y	Y	Y	Y	Y	N/A	Y
Ng et al. (19)	Y	Y	Y	N	N/A	Y	Y	Y	N	Y	Y
Tan et al. (22)	Y	Y	Y	N	Y	Y	Y	Y	Y	Y	Y
Ligezka et al. (18)	Y	Y	Y	U	N	Y	Y	Y	N	Y	Y
Barbosa and Vieira-Coelho (16)	Y	Y	Y	N	Y	Y	N/A	Y	U	Y	Y
Alanazi (15)	Y	Y	U	N	N/A	Y	Y	Y	N	N/A	Y
Present study	Y	Y	Y	Y	Y	Y	Y	Y	N	Y	Y

Q1, Is the evaluation question unambiguous?; Q2, Were there sufficient inclusion criteria to answer the research question?; Q3, Was the search strategy appropriate?; Q4, Were there insufficient means or sources used to find studies?; Q5, Were the study-evaluation standards adequate?; Q6, Did at least two separate reviewers each make their own critical judgments?; Q7, Was there a way to reduce human error during data collection?; Q8, Were the strategies for combining studies adequate?; Q9, Was the potential for bias in the publication process evaluated?; Q10, Were the reported data sufficient to back up the suggested changes to policy and/or practice?; Q11, Were the detailed instructions for new studies adequate?

However, upon closer examination, we identified certain biases in all the systematic reviews that were included. Recurring worries included the use of predominant studies that compared all patients to the same standard test of nutritional intervention. This approach raised questions about potential bias.

## Discussion

This systematic review and meta-analysis was aimed to assess the efficacy and safety of psychobiotics in ASD subjects, and show that those there was no significant effect of such therapy on autism-related behavioral symptoms, it has significant effect on the brain connectivity through frontopolar power in beta and gamma bands mediated by chemicals and cytokines, such as *TNF*-α. The psychobiotics showed no serious side-effects.

ASD represents a neurodevelopmental condition marked by enduring deficits in social interaction and communication. Alongside these challenges are repetitive and restricted behavior patterns, interests, or activities. The complexities and obstacles associated with ASD result from a combination of factors and manifest through a wide range of symptoms, encompassing issues like impaired social interactions, communication difficulties, and repetitive behaviors. The increasing prevalence of autism spectrum disorder highlights the urgent need to implement effective therapies all over the world.

The current understanding is that ASD arises from a complex interplay between environmental and genetic influences. Several variables have been identified as contributing to developing problems with the immune system and genetic structure (4, 5, 57–59). The study conducted by Malkova et al. (5) observed an increase in the risk of autism spectrum disorder in children whose mothers experienced immunological activation during pregnancy. The examination conducted in this context is noteworthy because it investigates the possible use of probiotics, prebiotics, and synbiotics as therapies. The intricate relationship between gut wellness and neurological problems is the focus of the article.

The study’s results are supported by reputable sources, including Schmitt et al. (40) and Kang et al. (7), which enhances the study’s credibility and strengthens its overall validity. The present paper includes a comprehensive meta-analysis of randomized controlled trials (RCTs) examining the effect of probiotics, prebiotics, and synbiotics on symptoms associated with ASD. The results of these studies involve improvements in actions, gastrointestinal function, and general quality of life. Nevertheless, it is important to acknowledge that the findings are influenced by the intrinsic diversity in the research, which arises from differences in the protocols of the interventions and the characteristics of the participants.

According to our data, the behavioral symptoms associated with ASD do not improve between the beginning and end of treatment.

Children who were given probiotics had reduced frontopolar power, according to the study, than that of children who did not receive probiotics, while frontopolar power was higher. Subjects with their eyes open produce beta waves, which are linked to physiological activation, attention, concentration, analytical thought, and states of focused attention, deep thought, and full mental or motor engagement (60). Gamma waves are linked to early sensory reactions and working-memory tasks (61). The resting electroencephalogram (EEG) of people with autism spectrum disorder typically displays elevated activity in the delta, theta, beta, and gamma frequency bands (62–64). When it comes to distinguishing autistic disorder from other conditions, beta power is regarded as one of the finest indices, with a 95.2% accuracy rate (65).

Coherence increases after probiotic supplementation, and this is correlated with reduced levels of cytokines like *TNF*-α, according to an analysis of the relationships between EEG and biochemical measures. Levels of *TNF*-α, an inflammatory biomarker found in the brain and CSF of many autistic people, have been found to be positively correlated with the severity of autism spectrum disorders (66). Considering the importance of *TNF*-α in controlling highly functional and plasticity, it is clear that this protein has an effect on EEG patterns (67). This suggests that the chemicals, cytokines, and hormones secreted by the gut microbiota and influenced by probiotic administration may be mediating the alterations in brain connectivity that we described.

The incorporation of several age cohorts in the research contributes an enhanced level of complexity to its results. The research recognizes the dynamic character of autism spectrum disorder (ASD) and the possible variations in intervention outcomes depending on age, taking into account both preschool-aged children (39) and people across multiple stages of development (45). Because of the well-known connection between gut health and brain health, this article centers on the microbiome of the digestive tract (13). Many neurological and psychiatric disorders, particularly ASD, have been linked to this symbiotic interaction between the brain and the digestive system. The major goal of this study is to investigate therapies that affect this axis, highlighting its possible importance in delivering comprehensive care to people with ASD.

By conducting a meta-analysis and systematic review of the relevant literature, the paper provides a substantial contribution to our understanding of the potential benefits of probiotics, prebiotics, and synbiotics as additional therapy for people with ASD. In order to properly address the many complexities of ASD, the research offers a critical evaluation of the present state of affairs and highlights the need for more centralized research methodology to be used. As our knowledge of the microbiome-gut-brain axis expands, we anticipate that medicines supported by evidence that improve gut health will play an increasingly significant role in the management of ASD.

## Limitations

Strict eligibility requirements imposed by the study’s sponsor contributed to a relatively small sample size. Potentially illuminating splits by sex and GI dysfunction type were not possible due to the small sample size. Another is though successful blinding in double-blind RCTs is *crucial for minimizing bias*, however studies rarely report information about blinding. In double-blind RCTs of therapies in ASD, *blinding can be broken due to the apparent side effects*. It would appear that adequate allocation concealment is the more crucial indicator. Furthermore, many trials, especially those involving children, cannot be double-blinded. A standard premised on double blinding is not applicable, so those trials must be evaluated on their own merits. A third factor is the use of an insensitive anxiety scale that was chosen because it was thought to be ASD-specific.

## Conclusion

The published studies on psychobiotics in patients with ASD provide encouraging insights into the potential benefits of modulating the gut microbiota for symptom improvement. The results of this review shows that psychobiotics impose a medium effect on ASD-related symptoms. These interventions may hold promise as complementary or adjunct therapies for individuals with these neurodevelopmental disorders. Our results lend credence to the use of psychobiotics in a sizable population of people with ASD. The results of this pilot study also pave the way for future studies to use EEG activity as a quantitative objective marker of efficacy of treatment in children with ASD. However, further research, including larger and more controlled clinical trials, is necessary to better understand the mechanisms at play and to elaborate clear guidelines for their use in clinical practice.

## Data availability statement

The original contributions presented in the study are included in the article/Supplementary material, further inquiries can be directed to the corresponding author.

## Author contributions

FR: Conceptualization, Data curation, Formal analysis, Methodology, Project administration, Validation, Writing – original draft, Writing – review & editing. KT: Data curation, Investigation, Project administration, Resources, Writing – review & editing. NQ: Data curation, Investigation, Resources, Software, Writing – review & editing. KD: Conceptualization, Methodology, Project administration, Visualization, Writing – original draft, Writing – review & editing. AZ: Data curation, Formal analysis, Investigation, Software, Writing – original draft. RK: Data curation, Investigation, Validation, Visualization, Writing – review & editing.
