# Supplementary material for: Probiotics, prebiotics, and synbiotics for patients with autism spectrum disorder: a meta-analysis and umbrella review
Source: Front Nutr. 2023 Dec 11;10:1294089. doi: 10.3389/fnut.2023.1294089 (PMC10750421; doi:10.3389/fnut.2023.1294089)

**Probiotics, prebiotics, and synbiotics for**

**patients with autism spectrum disorder: A systematic review and meta-analysis of randomized controlled trials**

**Appendix 1**

| **Search number** | **Query** | **Results** |
| --- | --- | --- |
| 16 | ((((((autistic traits[Title/Abstract]) OR (Asperger disorder[Title/Abstract])) OR (Asperger syndrome[Title/Abstract])) OR (autistic disorder[Title/Abstract])) OR (ASD[Title/Abstract])) OR (autism[Title/Abstract])) OR (autism spectrum disorder[Title/Abstract]) | 1,627 |
| 15 | (nutrition[Title/Abstract]) OR (((((Probiotics[Title/Abstract]) OR (prebiotics[Title/Abstract])) OR (synbiotics[Title/Abstract])) OR (microbiome[Title/Abstract])) OR (microflora[Title/Abstract])) | 315,699 |
| 14 | ((((Probiotics[Title/Abstract]) OR (prebiotics[Title/Abstract])) OR (synbiotics[Title/Abstract])) OR (microbiome[Title/Abstract])) OR (microflora[Title/Abstract]) | 97,426 |
| 13 | microflora[Title/Abstract] | 18,456 |
| 12 | microbiome[Title/Abstract] | 55,907 |
| 11 | autistic traits[Title/Abstract] | 1,683 |
| 10 | Asperger disorder[Title/Abstract] | 94 |
| 9 | Asperger syndrome[Title/Abstract] | 1,127 |
| 8 | autistic disorder[Title/Abstract] | 1,542 |
| 7 | ASD[Title/Abstract] | 34,940 |
| 6 | autism[Title/Abstract] | 62,067 |
| 5 | autism spectrum disorder[Title/Abstract] | 27,710 |
| 4 | nutrition[Title/Abstract] | 222,149 |
| 3 | synbiotics[Title/Abstract] | 1,655 |
| 2 | prebiotics[Title/Abstract] | 6,336 |
| 1 | Probiotics[Title/Abstract] | 26,230 |

**Appendix 2**

| **Database** | **Query** | **Results** |
| --- | --- | --- |
| CNKI | (“autism spectrum disorder” OR “ASD” OR “autism” OR “autistic disorder” OR “asperger syndrome” OR “asperger disorder” OR “autistic traits”) AND  (“microbiota” OR “microbiome” OR “microflora” OR “probiotic” OR “probiotics”) | 15 |
| PubMed | ((((((autistic traits[Title/Abstract]) OR (Asperger disorder[Title/Abstract])) OR (Asperger syndrome[Title/Abstract])) OR (autistic disorder[Title/Abstract])) OR (ASD[Title/Abstract])) OR (autism[Title/Abstract])) OR (autism spectrum disorder[Title/Abstract]) | 1,627 |
| Web of  Science | (“microbiota” OR “microbiome” OR “microflora” OR “probiotic” OR  “probiotics”). Then, use its built-in filtering function in web of science to filter out studies with  study types “Abstract”, “Meeting”, “News”, “Letter”, “Biography”, “Book”, “Correction”,  “Unspecified” , “Editorial Material”, “Case Report”, “Early Acess”, “Other”, “Review Article | 1,254 |
| Scopus | ((((((autistic traits[Title/Abstract]) OR (Asperger disorder[Title/Abstract])) OR (Asperger syndrome[Title/Abstract])) OR (autistic disorder[Title/Abstract])) OR (ASD[Title/Abstract])) OR (autism[Title/Abstract])) OR (autism spectrum disorder[Title/Abstract]) | 476 |
| Cochrane  library | “autism spectrum disorder” AND "Probiotics" OR "prebiotics" OR "synbiotics" | 21 |

**Appendix 3**

| **Section and Topic** | **Item #** | **Checklist item** | **Location where item is reported** |
| --- | --- | --- | --- |
| **TITLE** | | |  |
| Title | 1 | Identify the report as a systematic review. | Search methods |
| **ABSTRACT** | | |  |
| Abstract | 2 | See the PRISMA 2020 for Abstracts checklist. |  |
| **INTRODUCTION** | | |  |
| Rationale | 3 | Describe the rationale for the review in the context of existing knowledge. | Introduction paragraph 4 |
| Objectives | 4 | Provide an explicit statement of the objective(s) or question(s) the review addresses. | Introduction last paragraph |
| **METHODS** | | |  |
| Eligibility criteria | 5 | Specify the inclusion and exclusion criteria for the review and how studies were grouped for the syntheses. | Eligibility criteria |
| Information sources | 6 | Specify all databases, registers, websites, organisations, reference lists and other sources searched or consulted to identify studies. Specify the date when each source was last searched or consulted. | Search methods |
| Search strategy | 7 | Present the full search strategies for all databases, registers and websites, including any filters and limits used. | Appendix |
| Selection process | 8 | Specify the methods used to decide whether a study met the inclusion criteria of the review, including how many reviewers screened each record and each report retrieved, whether they worked independently, and if applicable, details of automation tools used in the process. | Screening |
| Data collection process | 9 | Specify the methods used to collect data from reports, including how many reviewers collected data from each report, whether they worked independently, any processes for obtaining or confirming data from study investigators, and if applicable, details of automation tools used in the process. | Data extraction and synthesis |
| Data items | 10a | List and define all outcomes for which data were sought. Specify whether all results that were compatible with each outcome domain in each study were sought (e.g. for all measures, time points, analyses), and if not, the methods used to decide which results to collect. | Eligibility criteria  Data extraction and synthesis |
|  | 10b | List and define all other variables for which data were sought (e.g. participant and intervention characteristics, funding sources). Describe any assumptions made about any missing or unclear information. | Characteristics of studies table |
| Study risk of bias assessment | 11 | Specify the methods used to assess risk of bias in the included studies, including details of the tool(s) used, how many reviewers assessed each study and whether they worked independently, and if applicable, details of automation tools used in the process. | Risk of bias assessment |
| Effect measures | 12 | Specify for each outcome the effect measure(s) (e.g. risk ratio, mean difference) used in the synthesis or presentation of results. | Data extraction and synthesis |
| Synthesis methods | 13a | Describe the processes used to decide which studies were eligible for each synthesis (e.g. tabulating the study intervention characteristics and comparing against the planned groups for each synthesis (item #5)). | Data extraction and synthesis |
|  | 13b | Describe any methods required to prepare the data for presentation or synthesis, such as handling of missing summary statistics, or data conversions. | NA |
|  | 13c | Describe any methods used to tabulate or visually display results of individual studies and syntheses. | - |
|  | 13d | Describe any methods used to synthesize results and provide a rationale for the choice(s). If meta-analysis was performed, describe the model(s), method(s) to identify the presence and extent of statistical heterogeneity, and software package(s) used. | Data extraction and synthesis |
|  | 13e | Describe any methods used to explore possible causes of heterogeneity among study results (e.g. subgroup analysis, meta-regression). | - |
|  | 13f | Describe any sensitivity analyses conducted to assess robustness of the synthesized results. | - |
| Reporting bias assessment | 14 | Describe any methods used to assess risk of bias due to missing results in a synthesis (arising from reporting biases). | Funnel plot performed (but not described due to word count. Detail available on request) |
| Certainty assessment | 15 | Describe any methods used to assess certainty (or confidence) in the body of evidence for an outcome. | GRADE was used, but not described (due to word count. Detail available on request) |
| **RESULTS** | | |  |
| Study selection | 16a | Describe the results of the search and selection process, from the number of records identified in the search to the number of studies included in the review, ideally using a flow diagram. | Study selection and PRISMA flowchart |
|  | 16b | Cite studies that might appear to meet the inclusion criteria, but which were excluded, and explain why they were excluded. | PRISMA flowchart for number of studies excluded with reasons. Authors can be contacted for full list of citations. |
| Study characteristics | 17 | Cite each included study and present its characteristics. | Table 1 |
| Risk of bias in studies | 18 | Present assessments of risk of bias for each included study. | Table 1 |
| Results of individual studies | 19 | For all outcomes, present, for each study: (a) summary statistics for each group (where appropriate) and (b) an effect estimate and its precision (e.g. confidence/credible interval), ideally using structured tables or plots. | Figure 2 and Figure 3 |
| Results of syntheses | 20a | For each synthesis, briefly summarise the characteristics and risk of bias among contributing studies. | Risk of bias within and across studies |
|  | 20b | Present results of all statistical syntheses conducted. If meta-analysis was done, present for each the summary estimate and its precision (e.g. confidence/credible interval) and measures of statistical heterogeneity. If comparing groups, describe the direction of the effect. | Figure 2 and Figure 3  Results synthesis |
|  | 20c | Present results of all investigations of possible causes of heterogeneity among study results. | Discussion of high risk of bias |
|  | 20d | Present results of all sensitivity analyses conducted to assess the robustness of the synthesized results. | NA |
| Reporting biases | 21 | Present assessments of risk of bias due to missing results (arising from reporting biases) for each synthesis assessed. | NA |
| Certainty of evidence | 22 | Present assessments of certainty (or confidence) in the body of evidence for each outcome assessed. | Not presented due to word count. Detail available on request |
| **DISCUSSION** | | |  |
| Discussion | 23a | Provide a general interpretation of the results in the context of other evidence. | Discussion paragraph 1 and 2 |
|  | 23b | Discuss any limitations of the evidence included in the review. | Discussion paragraph 2 and 3 |
|  | 23c | Discuss any limitations of the review processes used. | Discussion paragraph 7 |
|  | 23d | Discuss implications of the results for practice, policy, and future research. | Conclusion |
| **OTHER INFORMATION** | | |  |
| Registration and protocol | 24a | Provide registration information for the review, including register name and registration number, or state that the review was not registered. | Protocol was not registered (statement removed due to word count) but selection criteria were signed off a priori (under ‘protocol’) |
|  | 24b | Indicate where the review protocol can be accessed, or state that a protocol was not prepared. | NA |
|  | 24c | Describe and explain any amendments to information provided at registration or in the protocol. | No amendments |
| Support | 25 | Describe sources of financial or non-financial support for the review, and the role of the funders or sponsors in the review. | Funding |
| Competing interests | 26 | Declare any competing interests of review authors. | Conflicts of Interest |
| Availability of data, code and other materials | 27 | Report which of the following are publicly available and where they can be found: template data collection forms; data extracted from included studies; data used for all analyses; analytic code; any other materials used in the review. | NA |

Subgroup analysis

Country-wised analysis


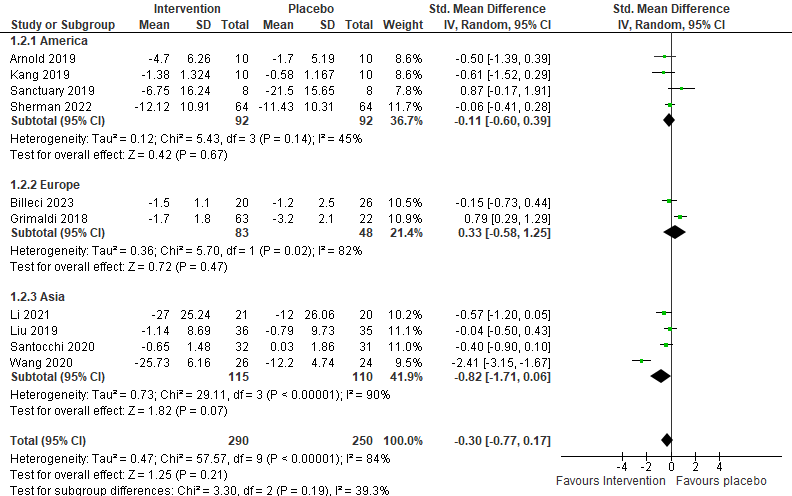


Nutritional type


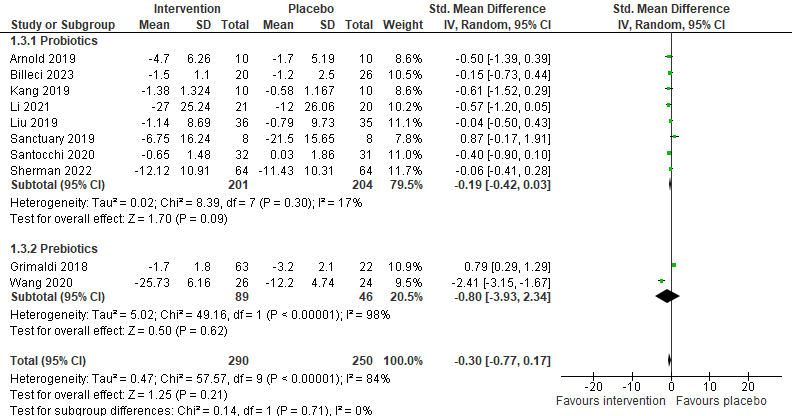

Supplement: Supplementary file 1 [file Data_Sheet_1.docx]
